# Validated method for measuring airborne concentrations of *tert*-butylphenols for occupational exposure assessment

**DOI:** 10.1093/joccuh/uiag027

**Published:** 2026-05-14

**Authors:** Akito Takeuchi, Ai Yamada, Tomiko Tashiro, Maika Inoue, Yuriko Miyama, Ryoko Imoto, Kyoko Adachi, Shinobu Yamamoto, Yoko Endo, Ginji Endo

**Affiliations:** Kinki Osaka Safety and Health Service Center, Japan Industrial Safety and Health Association, 2-3-8 Tosabori, Nishi-Ku, Osaka 550-0001, Japan; Occupational Health Research and Development Center, Japan Industrial Safety and Health Association, 5-35-2 Shiba, Minato-Ku, Tokyo 108-0014, Japan; Kinki Osaka Safety and Health Service Center, Japan Industrial Safety and Health Association, 2-3-8 Tosabori, Nishi-Ku, Osaka 550-0001, Japan; Department of Pathophysiological Laboratory Sciences, Nagoya University Graduate School of Medicine, 1-1-20 Daiko-minami, Higashi-Ku, Nagoya 461-8673, Japan; Kinki Osaka Safety and Health Service Center, Japan Industrial Safety and Health Association, 2-3-8 Tosabori, Nishi-Ku, Osaka 550-0001, Japan; Kinki Osaka Safety and Health Service Center, Japan Industrial Safety and Health Association, 2-3-8 Tosabori, Nishi-Ku, Osaka 550-0001, Japan; Kinki Osaka Safety and Health Service Center, Japan Industrial Safety and Health Association, 2-3-8 Tosabori, Nishi-Ku, Osaka 550-0001, Japan; Kinki Osaka Safety and Health Service Center, Japan Industrial Safety and Health Association, 2-3-8 Tosabori, Nishi-Ku, Osaka 550-0001, Japan; Kinki Osaka Safety and Health Service Center, Japan Industrial Safety and Health Association, 2-3-8 Tosabori, Nishi-Ku, Osaka 550-0001, Japan; Research Center for Safety Science, Nagoya University, Furo-cho, Chikusa-ku, Nagoya 464-8601, Japan; Kinki Osaka Safety and Health Service Center, Japan Industrial Safety and Health Association, 2-3-8 Tosabori, Nishi-Ku, Osaka 550-0001, Japan; Kinki Osaka Safety and Health Service Center, Japan Industrial Safety and Health Association, 2-3-8 Tosabori, Nishi-Ku, Osaka 550-0001, Japan

**Keywords:** air sampling method, high-performance liquid chromatography, *tert*-butylphenol, personal exposure measurement, workplace air

## Abstract

**Objectives:**

This study aimed to develop a method for measuring airborne concentrations of *tert*-butylphenols (*tert*-BPs) to assess workers' occupational exposure.

**Methods:**

Air samples were collected using an XAD-7 sorbent tube. *tert*-BPs were desorbed from the sorbent with methanol, and their concentrations in the desorbed solutions were determined via high-performance liquid chromatography with fluorescence detection. Method validation included evaluation of retention efficiency, storage stability, method quantitation limit, and reproducibility.

**Results:**

The retention efficiencies for 2-*tert*-BP, 3-*tert*-BP, and 4-*tert*-BP in the XAD-7 sorbent tube were 89%-97%, 93%-96%, and 95%-96%, respectively. Relative standard deviations, reflecting the overall reproducibility of the proposed method, ranged from 0.5% to 5.9% for all *tert*-BPs. *tert*-BPs remained stable on the XAD-7 sorbent tube for at least 7 days at 4°C. The method quantitation limit was 0.118 μg/sample for each *tert*-BP.

**Conclusions:**

The developed method provides a robust and reproducible approach for assessing occupational exposure to airborne *tert*-BPs, enabling accurate determination within a concentration range of 0.8-160 ppb. This method can facilitate more precise risk assessments in occupational environments.

## Teaser

**Key points**
 

- **What is already known on this topic:**  *tert*-Butylphenols (*tert*-BPs) are used as a raw material for various products, including agrochemicals, fragrances, phenolic resins, ultraviolet absorbers, and surfactants. The Deutsche Forschungsgemeinschaft (DFG, German Research Foundation) has proposed an occupational exposure limit for 4-*tert*-BP, due to its adverse health effects. However, no validated method currently exists for assessing worker exposure to airborne *tert*-BPs.
- **What this study adds:** This study presents a validated method utilizing an XAD-7 sorbent tube as the sampling medium, enabling measurement of personal exposure concentrations of *tert*-BPs ranging from 0.8 to 160 ppb in a 4-hour sampling period.
- **How this study might affect research, practice, or policy:** The developed method provides a means for estimating worker exposure to *tert*-BPs and can support improved monitoring and prevention of occupational health risks associated with these compounds.

## 1. Introduction

*tert*-Butylphenols (*tert*-BPs) exist as 3 positional isomers: 2-*tert*-BP (CAS RN 88-18-6), 3-*tert*-BP (CAS RN 585-34-2), and 4-*tert*-BP (CAS RN 98-54-4). They are used as raw materials in agrochemicals, fragrances, phenolic resins (including oil-soluble types), ultraviolet absorbers, and surfactants, and also serve as modifiers for phenolic resins.^1^

In Japan, employers are required to conduct risk assessments for workers engaged in the manufacture or handling of 2-*tert*- and 4-*tert*-BPs, in accordance with Article 57-3 of the Industrial Safety and Health Act.^2^ Although neither the Japan Society for Occupational Health nor the American Conference of Governmental Industrial Hygienists (ACGIH) have established an occupational exposure limit (OEL) for *tert*-BPs, the Deutsche Forschungsgemeinschaft (DFG)^3^^,^^4^ has proposed an OEL of 0.08 ppm (Maximale Arbeitsplatzkonzentrationen, MAK) only for 4-*tert*-BP, noting that “this substance can occur simultaneously as vapor and aerosol.” However, to the best of our knowledge, no methods have been reported for measuring airborne concentrations of *tert*-BPs in occupational settings. Therefore, the aim of this study was to establish a reliable and practical method for assessing worker exposure to airborne *tert*-BPs.

## 2. Materials and methods

### 2.1 Materials

2-*tert*-, 3-*tert*-, 4-*tert*-, 2-*sec*-, and 4-*sec*-BPs were purchased from Tokyo Chemical Industry. High-performance liquid chromatography (HPLC)–grade methanol was obtained from Kanto Chemical Co, Inc. Ultrapure water was produced using a PURELAB flex-3 system (Organo Corp). A mixed standard stock solution of *tert*-BPs (each at 4720 μg/mL) was prepared in methanol and stored at 4°C. An XAD-7 sorbent tube (100 mg/50 mg, Catalog No. 226-95) was purchased from SKC Inc.

### 2.2 Method validation procedure

Method development and validation followed the guidelines of the National Institute for Occupational Safety and Health (NIOSH)^5^ and the US Department of Labor, Occupational Safety and Health Administration (OSHA).^6^ The evaluation parameters included retention efficiency, storage stability, method quantitation limit, and reproducibility. For the retention efficiency and storage stability tests, XAD-7 sorbent tubes were spiked with 5-μL aliquots of *tert*-BP standard solutions (23.6–4720 μg/mL) onto the front glass wool plug of each tube. Room air (temperature 20.0°C-25.9°C; relative humidity 25%-62%) was then drawn through each tube using an automatic air sampling pump (GSP-311FT, GASTEC Corp) at a flow rate of 0.1 L/min for 240 minutes. For the storage stability test, after air sampling, the tubes were capped and stored in a refrigerator (4°C) for 7 days. Five different spiked amounts were used for the retention efficiency test and 3 for the storage stability test, corresponding to approximately 0.8-160 ppb—representing roughly 1/100 to 2 times the MAK value proposed by DFG. To construct calibration curves, 8 concentrations (0.0295-11.8 μg/mL) were prepared by diluting the *tert*-BP-mixed standard stock solution with methanol. Then, the peak areas obtained via HPLC analysis were plotted against the corresponding concentrations of each *tert*-BP.

### 2.3 Analytical procedure and instrumental conditions

After passing room air through the spiked XAD-7 sorbent tubes, each section was transferred to separate glass test tubes. The front glass wool plug and its corresponding front sorbent section were placed together in the same test tube. Methanol (2 mL) was added to each tube, followed by sonication for 30 minutes to desorb *tert*-BPs. The desorbed solution was filtered through a DISMIC-13HP020AN filter (Advantec Toyo Kaisha, Ltd) and placed in an autosampler vial for analysis. HPLC analysis was performed using a Prominence UFLC system equipped with an RF-20AXS fluorescence detector (FLD) (Shimadzu Corp). Chromatographic separation was achieved under the following conditions: column, COSMOSIL 3πNAP (150 × 3.0 mm internal diameter, 3 μm particle size; Nacalai Tesque Inc); flow rate = 0.6 mL/min; temperature = 40°C; mobile phase = ultrapure water:methanol (55:45, v/v); elution mode = isocratic elution; FLD excitation/emission wavelengths = 275 nm/300 nm; injection volume = 5 μL.

## 3. Results

To identify an analytical column suitable for separating the 5 BP isomers (2-*tert*-, 3-*tert*-, 4-*tert*-, 2-*sec*-, and 4-*sec*-BP), 4 different analytical columns were tested: COSMOSIL 3πNAP, InertSustain Phenyl HP, InertSustain C18 HP, and InertSustainSwift C18 HP (GL Sciences Inc), all with a 3-μm particle size and dimensions of 3.0 × 150 mm. Chromatograms obtained using each column are presented in Figure S1. Except for the InertSustain Phenyl HP, which was operated at 25°C, all other HPLC conditions were kept constant (see Materials and methods: Analytical procedure and instrumental conditions). The COSMOSIL 3πNAP column provided the best separation performance and was therefore selected for subsequent analyses. Spectral analysis indicated that the optimal excitation and emission wavelengths were 275 and 300 nm, respectively (Figure S2). These wavelengths were used for subsequent analyses.

**Figure 1:**
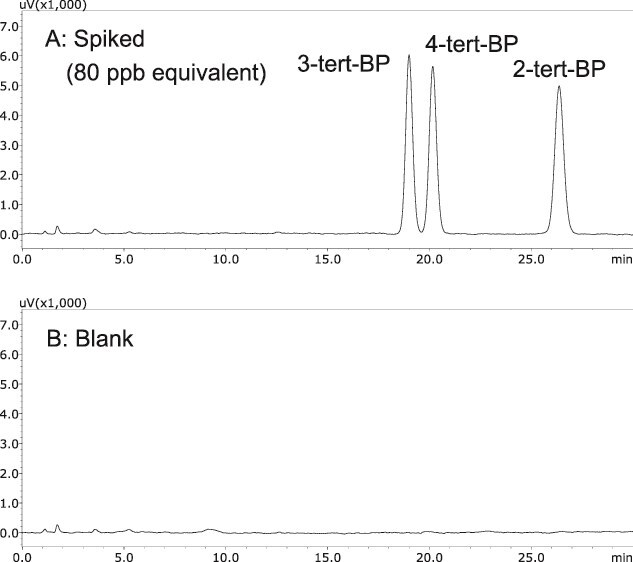
Chromatograms of solutions extracted from the front section of the XAD-7 sorbent tube after passing 24 L of room air through tubes either (A) spiked with 11.8 μg of each *tert*-BP (equivalent to 80 ppb each) or (B) left unspiked.

The validation results for the developed method are summarized in Table 1. The retention efficiencies were 89%-97%, 93%-96%, and 95%-96% for 2-*tert*-BP, 3-*tert*-BP, and 4-*tert*-BP, respectively. Relative standard deviations (RSDs), representing the overall reproducibility of the proposed method, were calculated from the obtained retention efficiencies with ranges of 0.8%-2.9%, 0.5%-4.4%, and 0.6%-5.9% for 2-*tert*-BP, 3-*tert*-BP, and 4-*tert*-BP, respectively. No *tert*-BPs were detected in the back sorbent section of any spiked XAD-7 sorbent tube during the retention efficiency test, indicating no breakthrough. Chromatograms of the desorbed solutions after passing 240 L of room air through the XAD-7 sorbent tube—with and without *tert*-BP spiking—are shown in Figure 1A and 1B, respectively; no *tert*-BPs are detected in Figure 1B. The storage rates were calculated by comparing the *tert*-BP amounts in the XAD-7 sorbent tubes before and after storage. The storage rate for each *tert*-BP exceeded 97% after 7 days of storage in a refrigerator (4°C). The calibration curves for each *tert*-BP were linear between 0.0295 and 11.8 μg/mL, with coefficients of determination (*R*^2^) above 0.999. The instrumental limits of quantitation for each *tert*-BP, calculated as 10 times the SD (*n* = 5) of the peak area for the lowest standard solution concentration (0.0295 μg/mL) divided by the slope of the calibration curve and multiplied by the desorption volume (2 mL), were 0.0246, 0.0292, and 0.0615 μg/sample for 2-*tert*-BP, 3-*tert*-BP, and 4-*tert*-BP, respectively. The method limit of quantitation, defined as the smallest spiked amount of *tert*-BP yielding >90% retention efficiency within the test range, was 0.118 μg/sample for each *tert*-BP.

**Table 1:** Retention efficiency and storage stability tests.^a^

Spiked amount, μg	Retention efficiency		Storage rate (%, *n* = 5)									
	(%, *n* = 5)		0 days		1 day		3 days		5 days		7 days	
	Mean ± SD	RSD	Mean ± SD	RSD	Mean ± SD	RSD	Mean ± SD	RSD	Mean ± SD	RSD	Mean ± SD	RSD
**2-*tert*-BP**												
** 0.118**	89 ± 2.6	2.9	100 ± 2.2	2.2	98 ± 0.7	0.7	95 ± 1.6	1.7	96 ± 5.6	5.8	97 ± 2.8	2.9
** 0.590**	95 ± 1.3	1.4	—	—	—	—	—	—	—	—	—	—
** 1.18**	94 ± 1.3	1.3	100 ± 1.3	1.3	100 ± 0.6	0.6	97 ± 2.2	2.3	98 ± 1.0	1.0	98 ± 2.5	2.5
** 11.8**	97 ± 0.8	0.8	—	—	—	—	—	—	—	—	—	—
** 23.6**	95 ± 2.3	2.4	100 ± 2.0	2.0	100 ± 0.9	0.9	102 ± 1.4	1.4	100 ± 0.5	0.5	101 ± 1.1	1.1
**3-*tert*-BP**												
** 0.118**	93 ± 4.1	4.4	100 ± 4.6	4.6	96 ± 2.6	2.7	96 ± 2.3	2.4	100 ± 3.2	3.2	99 ± 4.5	4.6
** 0.590**	95 ± 1.5	1.5	—	—	—	—	—	—	—	—	—	—
** 1.18**	95 ± 0.9	1.0	100 ± 1.4	1.4	101 ± 0.8	0.8	97 ± 0.7	0.8	98 ± 1.7	1.8	98 ± 3.6	3.7
** 11.8**	96 ± 0.5	0.5	—	—	—	—	—	—	—	—	—	—
** 23.6**	95 ± 2.4	2.5	100 ± 2.1	2.1	100 ± 1.0	1.0	103 ± 1.4	1.4	100 ± 0.6	0.5	101 ± 1.2	1.1
**4-*tert*-BP**												
** 0.118**	96 ± 5.7	5.9	100 ± 2.3	2.3	99 ± 1.8	1.8	100 ± 5.7	5.7	98 ± 4.0	4.1	101 ± 3.5	3.5
** 0.590**	96 ± 1.5	1.5	—	—	—	—	—	—	—	—	—	—
** 1.18**	95 ± 1.0	1.0	100 ± 1.4	1.4	101 ± 1.0	1.0	97 ± 0.7	0.7	99 ± 1.2	1.2	97 ± 3.7	3.8
** 11.8**	95 ± 0.5	0.6	—	—	—	—	—	—	—	—	—	—
** 23.6**	95 ± 2.5	2.6	100 ± 1.9	1.9	100 ± 1.0	1.0	103 ± 1.4	1.4	100 ± 0.5	0.5	101 ± 1.2	1.2

Abbreviations: BP, butyl phenol; RSD, relative standard deviation.

^a^For the retention efficiency test, room air (temperature 20.0°C-25.9°C; relative humidity 25%-62%) was drawn through the *tert*-BP-spiked XAD-7 sorbent tube using the sampling pump at a flow rate of 0.1 L/min for 4 hours. For the storage stability test, after having room air drawn through them, the XAD-7 sorbent tubes were sealed and then stored in a refrigerator (4°C) for 7 days. The spiked amounts correspond to air concentrations of approximately 0.8-160 ppb.

## 4. Discussion

The OEL (0.08 ppm, as MAK) proposed by DFG^3^^,^^4^ for 4-*tert*-BP includes an endnote stating that “this substance can occur simultaneously as vapor and aerosol.” Similar endnotes for chemical substances, with saturated vapor concentration-to-OEL ratios typically between 0.1 and 10, have also been included by the ACGIH^7^ and the Ministry of Health, Labour and Welfare.^8^ Risk assessment of such substances requires the measurement of both particle and vapor phases. The saturated vapor pressures of 2-*tert*-, 3-*tert*-, and 4-*tert*-BP at 25°C, as obtained from CAS SciFinder (CAS, Columbus, OH, United States) and calculated using Advanced Chemistry Development (ACD/Labs) software, were 0.0740, 0.0251, and 0.0361 torr, respectively. Based on these values, the corresponding saturated vapor concentrations were estimated to be approximately 97, 33, and 48 ppm, respectively, yielding ratios of saturated vapor concentration to the OEL—calculated by applying an OEL of 0.08 ppm proposed by DFG for 4-*tert*-BP to the other 2 *tert*-BPs—of approximately 1217, 413, and 594, respectively. Because these ratios were well above 10, *tert*-BPs were considered to exist predominantly in the vapor phase under occupational conditions. Accordingly, the target analytes in this study were limited to vapor-phase *tert*-BPs. As solid sorbent tubes are suitable for sampling vapor-phase substances, this method was adopted in this study.

To the best of our knowledge, measurement methods for determining airborne *tert*-BPs, including workplace air, have not yet been reported. However, several methods for determining airborne concentrations of phenol and cresols, which are structurally similar to *tert*-BPs, have been reported.^9^^,^^10^ These methods employ an XAD-7 sorbent tube as the sampling medium and use either gas chromatography with flame ionization detection (GC-FID)^9^ or HPLC with ultraviolet detection (HPLC-UV).^10^ Building upon these approaches, we developed a method for *tert*-BPs using the XAD-7 sorbent tube as the sampling medium and HPLC-FLD as the analytical technique, which offers higher selectivity and sensitivity than GC-FID or HPLC-UV. Optimization of the HPLC-FLD analytical conditions was initially planned to separate and quantify multiple BP isomers—including *tert*-, *sec*-, *n*-, and *iso*-isomers (a total of 12 isomers)—considering their potential coexistence in workplace environments. However, such an optimization process could not be implemented because of the commercial unavailability and exorbitant costs of most of the isomers. Therefore, 5 obtainable isomers (2-*tert*-, 3-*tert*-, 4-*tert*-, 2-*sec*-, and 4-*sec*-BP) were selected as target analytes in this study. Among the 4 tested columns, the COSMOSIL 3πNAP column demonstrated the best performance in terms of resolution, peak shape, and analysis time, emerging as a suitable candidate for the analysis of the tested BP isomers.

The validation results confirmed that the proposed sampling method is robust and reproducible. Furthermore, *tert*-BPs collected in the XAD-7 sorbent tube remained stable for at least 7 days when stored at 4°C, suggesting practicality for analytical operations. The measurable concentration range of *tert*-BPs using this method was 0.8-160 ppb over a 4-hour sampling period with a sampling air volume of 24 L, corresponding to approximately 1/100 to 2 times the MAK value for 4-*tert*-BP.

A major limitation of this study is that the sorbent tubes were spiked with *tert*-BP standard solutions rather than vapors, since continuous generation of standard vapors at known ppb-level concentrations was not feasible. Although this approach is commonly used in method development for air sampling, it remains a significant methodological constraint because it does not fully reproduce the adsorption behavior of vapor-phase *tert*-BPs on the sorbent material. In this approach, *tert*-BPs were gradually vaporized from the *tert*-BP standard solutions spiked onto the front glass wool plug of each sorbent tube during the air sampling and were subsequently adsorbed onto the sorbent. However, the vapor concentration generated from the spiked standard solution is not constant during the air sampling period. Consequently, the estimated sampling efficiency and breakthrough volume may differ from those obtained under real workplace sampling conditions, although the direction of this potential bias cannot be determined from the present data. In addition, this method is intended solely for the assessment of vapor-phase *tert*-BPs, and the suitability of XAD-7 sorbent tubes for capturing aerosol-phase *tert*-BPs remains unverified. Consequently, if vapors and aerosols coexist in occupational environments, as noted by DFG, *tert*-BP concentrations may be underestimated. Future studies should therefore evaluate sampling approaches applicable to mixed-phase conditions, such as combined filter–sorbent tube methods or impinger-based approaches. Moreover, 7 of the 12 BP isomers were not commercially available or prohibitively expensive, preventing their inclusion in this study. Chromatographic interference could occur if such compounds coelute with *tert*-BPs; however, this issue may be mitigated through further optimization of the chromatographic conditions.

## 5. Conclusions

To the best of our knowledge, this study presents the first validated method that enables reliable measurement of personal exposure to airborne *tert*-BPs within the concentration range of 0.8-160 ppb over a 4-hour sampling period—corresponding to 1/100 to 2 times the MAK value for 4-*tert*-BP proposed by DFG. This method provides a practical approach for accurate risk assessment and the development of effective preventive strategies for workers handling *tert*-BPs.

## Supplementary Material

JOH-2025-0373-BR_Supplementary_Figure_1_260429_uiag027

JOH-2025-0373-BR_Supplementary_Figure_2_260429_uiag027

## Data Availability

The data underlying this article are available from the corresponding author upon reasonable request.
