# Supplementary material for: Validated method for measuring airborne concentrations of tert-butylphenols for occupational exposure assessment
Source: J Occup Health. 2026 May 14;68(1):uiag027. doi: 10.1093/joccuh/uiag027 (PMC13251736; doi:10.1093/joccuh/uiag027)
Supplement: JOH-2025-0373-BR_Supplementary_Figure_1_260429_uiag027 [file joh-2025-0373-br_supplementary_figure_1_260429_uiag027.pptx]

## Slide 1
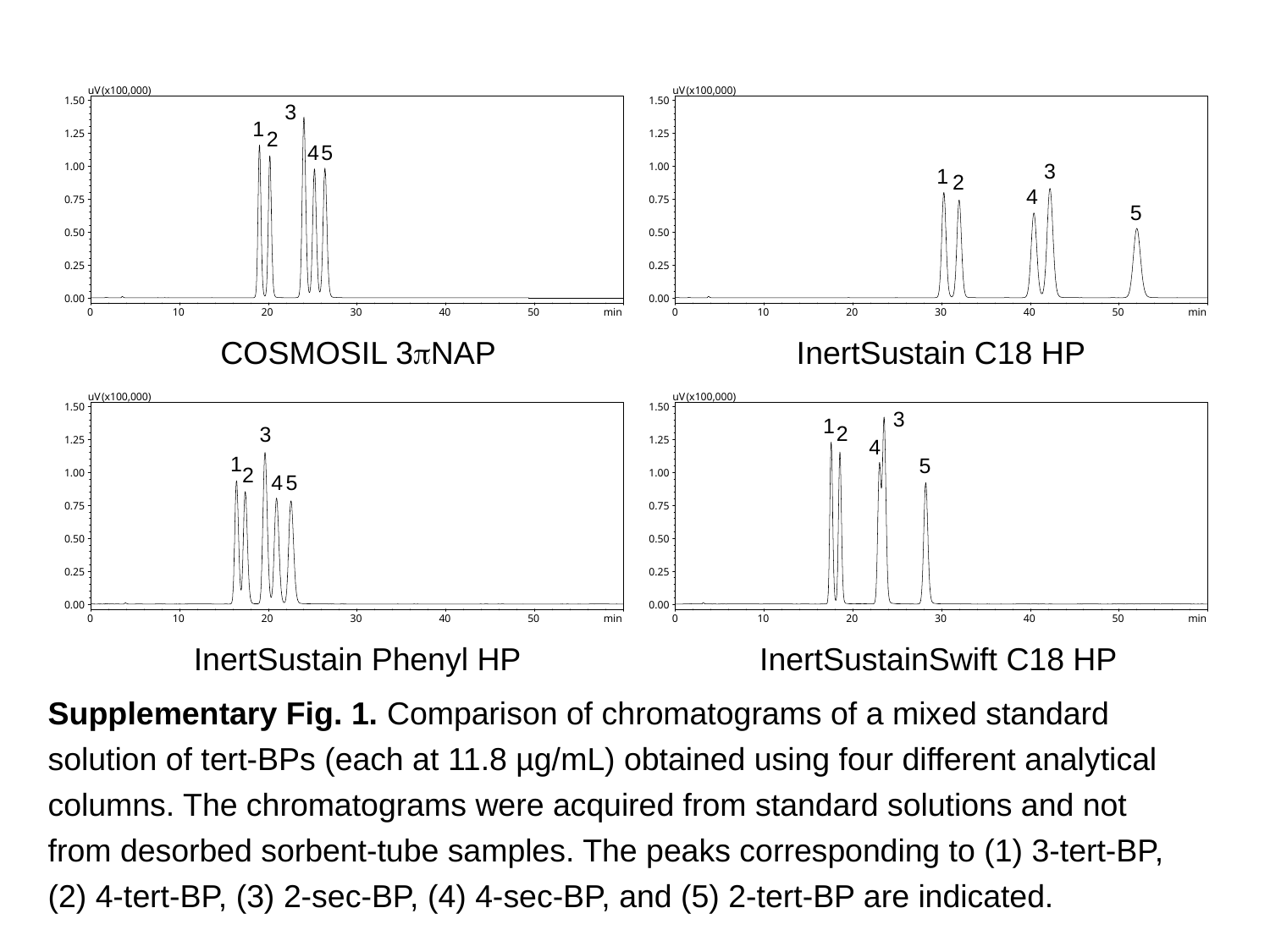

3
1
2
4 5
3
1
2
4
5
 COSMOSIL 3pNAP InertSustain C18 HP
3
1
2
3
4
1
5
2
4 5
 InertSustain Phenyl HP InertSustainSwift C18 HP
Supplementary Fig. 1. Comparison of chromatograms of a mixed standard solution of tert-BPs (each at 11.8 µg/mL) obtained using four different analytical columns. The chromatograms were acquired from standard solutions and not from desorbed sorbent-tube samples. The peaks corresponding to (1) 3-tert-BP, (2) 4-tert-BP, (3) 2-sec-BP, (4) 4-sec-BP, and (5) 2-tert-BP are indicated.
1
