# Supplementary material for: Validated method for measuring airborne concentrations of tert-butylphenols for occupational exposure assessment
Source: J Occup Health. 2026 May 14;68(1):uiag027. doi: 10.1093/joccuh/uiag027 (PMC13251736; doi:10.1093/joccuh/uiag027)
Supplement: JOH-2025-0373-BR_Supplementary_Figure_2_260429_uiag027 [file joh-2025-0373-br_supplementary_figure_2_260429_uiag027.pptx]

## Slide 1
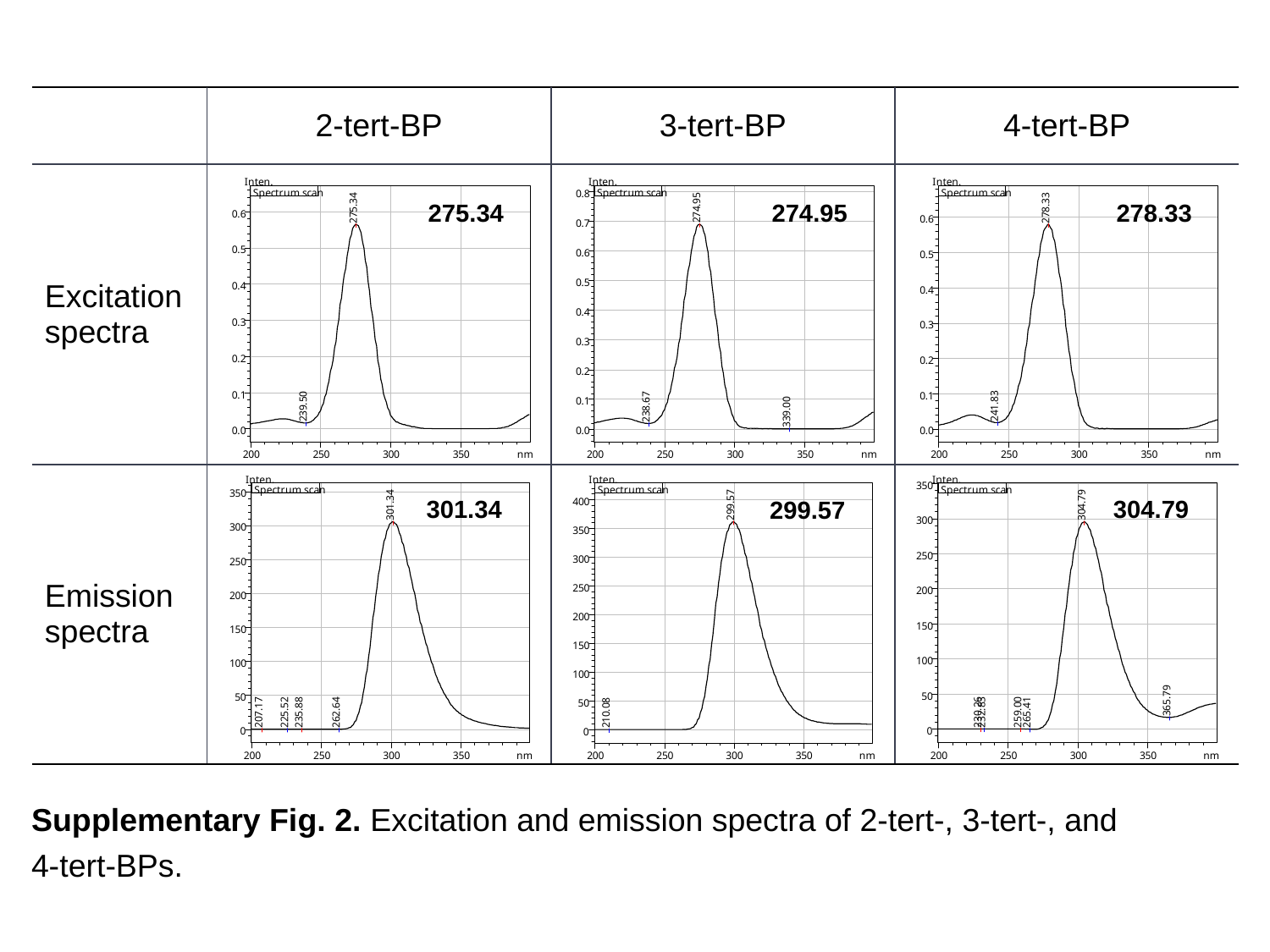

| | 2-tert-BP | 3-tert-BP | 4-tert-BP |
| --- | --- | --- | --- |
| Excitation spectra | | | |
| Emission spectra | | | |
275.34
274.95
278.33
301.34
299.57
304.79
Supplementary Fig. 2. Excitation and emission spectra of 2-tert-, 3-tert-, and 4-tert-BPs.
1
